# Native Mass Spectrometry of Soluble Proteins with Sodium Chloride Enabled by a Dendritic Detergent

**DOI:** 10.1021/acsmeasuresciau.6c00038

**Published:** 2026-06-09

**Authors:** Francesco Fiorentino, Wolf Hiller, Leonhard H. Urner

**Affiliations:** † Department of Biochemical Sciences, 9311 Sapienza University of Rome, Piazzale Aldo Moro 5, Rome 00185, Italy; ‡ Department of Chemistry and Chemical Biology, Technische Universität Dortmund, Otto-Hahn-Str. 6, Dortmund 44227, Germany

**Keywords:** native mass spectrometry, protein, lipid, electrospray, detergent, sodium chloride, semaglutide

## Abstract

Salts are essential for maintaining native protein structure and biomolecular interactions. Native mass spectrometry often yields uninterpretable spectra due to salt adduction and signal suppression. Existing workarounds require buffer exchange or extensive cleanup, which can disrupt weak noncovalent interactions and reduce throughput. Addressing this shortcoming, we establish a first-generation, dendritic triglycerol detergent as a simple-to-apply additive that enables native mass spectrometry of biophysically relevant protein concentrations in the presence of sodium chloride. Adding this detergent to sodium chloride–containing samples prior to electrospray ionization improves spectral quality, enhances signal-to-noise ratios, and enables an acquisition of interpretable native mass spectrometry data for soluble proteins, including complexes with lipids and drugs, at different ionic strengths. These improvements are achieved without compromising native-like charge-state distributions across proteins ranging from myoglobin and lipopolysaccharide transport protein H to bovine serum albumin and high-mass tetramers, such as alcohol dehydrogenase and pyruvate kinase. Nuclear magnetic resonance data indicate a concentration-dependent sodium chloride complexation by detergent in solution, consistent with desalted protein complexes observable in the gas phase. By rendering sodium chloride compatible with native mass spectrometry, first-generation, dendritic triglycerol detergent enables the routine analysis of protein complexes at otherwise inaccessible ionic strengths and biophysically relevant analyte concentrations.

## Graphical abstract

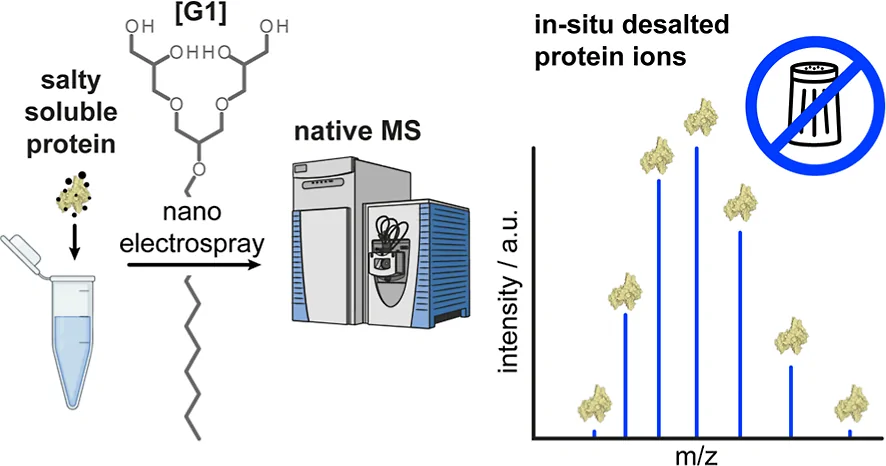

## Introduction

Sodium chloride (NaCl) is a kosmotropic salt that enhances hydrophobic interactions in water and protein–lipid complex stability in vivo.^1–3^ Native mass spectrometry (native MS) is increasingly used to study ligand binding, including the regulatory roles of lipids in protein structure and function.^4–6^ Because NaCl interferes with electrospray ionization, it is commonly displaced by volatile salts.^7^ A detailed analysis of salt-driven effects becomes impossible. Strategies enabling native MS of soluble proteins and biomolecular interactions at different ionic strengths are needed.

In native MS, samples are desolvated and ionized with electrospray ionization. To improve ion formation, nonvolatile salts are removed from samples by desalting techniques, like dialysis,^8,9^ chromatography,^10–12^ or diafiltration.^13^ These strategies facilitate the acquisition of native MS data but decrease the biophysical relevance of assay conditions, because salt is practically removed from samples prior to analysis.^7,14^ To enable native MS of samples containing biophysically relevant salts, in situ desalting strategies are being investigated. The idea is to maintain salt in solution and improve ionization with additives that reduce the relative intensity of sodium adducts detected by the mass analyzer. Established additives include ammonium salts,^15,16^ amino acids,^17,18^ or crown ethers.^19,20^ Supercharging reagents, like sulfolane or *m*-nitrobenzyl alcohol, can also improve ion intensity but promote a Coulomb-driven loss of noncovalent interactions.^21,22^ These advances highlight the need for sample additives that enable in situ desalting with compatibility across native MS workflows.

In this context, first-generation, dendritic triglycerol detergent [G1] represents a promising technology. Its outstanding compatibility with native MS compared to other detergent classes has been demonstrated for both soluble proteins^23^ and membrane proteins,^24–26^ including G protein-coupled receptors (GPCRs),^27–31^ and has recently been established in bottom-up proteomics.^32^ Aim of this work is to leverage recent advances in [G1] technology^33^ and native MS^34,35^ to evaluate its utility as an in situ desalting strategy, enabling the analysis of soluble proteins, including complexes with lipids and drugs, at biophysically relevant concentrations and ionic strengths.

## Experimental Section

### Chemicals and Model Protein Selection

To benchmark the utility of our in situ desalting strategy, we selected proteins differing in molecular mass and oligomeric state. The panel included myoglobin as a small monomeric protein, bovine serum albumin (BSA) as a larger monomer, lipopolysaccharide transport protein H (LptH) as a dimeric protein, and alcohol dehydrogenase (ADH) and pyruvate kinase as larger tetrameric assemblies. These proteins span an increasing native mass range, from myoglobin, approximately 17 kDa including the heme prosthetic group, to LptH, approximately 33 kDa as a dimer, BSA, approximately 66 kDa, ADH, approximately 147 kDa, and pyruvate kinase, approximately 232 kDa. This panel therefore allowed us to test whether [G1]-assisted desalting improves native MS across progressively larger and more complex protein systems, rather than in a single model protein. Unless otherwise specified, all chemicals were purchased from Sigma-Aldrich. The following proteins were also purchased from Sigma-Aldrich: myoglobin from horse skeletal muscle (Cat. n. M0630), BSA (Cat. n. A7030), ADH (Cat. n. A3263), pyruvate kinase from rabbit muscle (Cat. n. P9136). [G1] detergent was prepared as described previously.^33^

### Protein Expression and Purification

LptH was expressed and purified as described previously.^36^ Briefly, a modified pET-28a vector encoding an N-terminal 10 × His-tagged mature form of *Pseudomonas aeruginosa* LptH (residues 28–175), followed by a tobacco etch virus (TEV) protease cleavage site, was transformed into *E. coli* BL21­(DE3) (New England Biolabs). Cells were grown in LB medium supplemented with kanamycin (50 μg·mL^–1^) at 37 °C. At an OD_600_ of ∼0.6, expression was induced with isopropyl β-D-1-thiogalactopyranoside (final concentration 0.5 mM), and cultures were incubated for a further 16 h at 20 °C.

For protein purification, cell pellets were harvested by centrifugation (5,000*g*, 10 min, 4 °C) and resuspended in 250 mM NaCl, 25 mM Tris–HCl (pH 7.5), EDTA-free protease inhibitor cocktail (Roche), and DNase I (10 μg·mL^–1^). Cells were lysed by sonication, and insoluble material was removed by centrifugation (20,000*g*, 20 min, 4 °C). The clarified supernatant was filtered and loaded onto a 5 mL HisTrap HP column (Cytiva) equilibrated in 250 mM NaCl, 25 mM Tris–HCl (pH 7.5), and 20 mM imidazole. The column was washed with 50 mL of equilibration buffer, followed by 50 mL of 250 mM NaCl, 25 mM Tris–HCl (pH 7.5), and 80 mM imidazole, and protein was eluted in the same buffer containing 500 mM imidazole. Peak fractions were pooled, incubated with TEV protease, and dialyzed against 250 mM NaCl, 25 mM Tris–HCl (pH 7.5). The sample was reloaded onto a HisTrap HP column equilibrated in dialysis buffer; the flow-through containing cleaved LptH was collected, while His-tagged TEV and the cleaved His-tag peptide were retained on the resin. LptH was concentrated using an Amicon Ultra-15 (3 kDa MWCO; Millipore) to 6 mg·mL^–1^. Protein concentration was determined using an N50 NanoPhotometer (IMPLEN), and purity was assessed by SDS-PAGE (MES buffer) and mass spectrometry.

### Nuclear Magnetic Resonance Spectroscopy

To monitor interactions between [G1] and NaCl in solution, ^1^H NMR experiments were performed on the 600 MHz NMR spectrometer AVANCE NEO equipped with a helium-cooled triple resonance probe. The ^23^Na measurements were carried out at the 400 MHz NMR spectrometer AVANCE III HD with a 5 mm broadband probe. Both spectrometers are from Bruker BioSpin GmbH & Co. KG. ^1^H and ^23^Na DOSY measurements were measured of the sample solutions containing 10 mg of detergent and NaCl dissolved in deuterated water. The ^1^H DOSY measurements were performed using the 2D pulse sequence with the double stimulated echo for convection compensation and LED consisting of bipolar gradient pulses for diffusion and 3 spoil gradients.^37,38^ One hundred twenty-eight gradient amplitudes were linearly varied between 3 and 98% of the maximum gradient strength. Sixteen scans were acquired per gradient strength with an acquisition time of 2.78 s, a relaxation delay of 1.5 s and a spectrum width of 5882 Hz (9.8 ppm). In case of the ^23^Na DOSY, the stimulated echo sequence with LED and bipolar gradient pulses for diffusion using 2 spoil gradients were used.^39^ Sixty-four gradient amplitudes were linearly varied between 3 and 95% of the maximum gradient strength. Eight scans were recorded with an acquisition time of 0.56 s, a relaxation delay of 0.1 s and a spectrum width of 3644 Hz (33 ppm).

### Viscosity Analysis

To investigate whether changes in DOSY experiments reflect changes in sample viscosity, we determined kinematic viscosities using an Ubbhelode (Type 536 PC/I, SI Analytics) and a thermostat for (Haake DC 10, ThermoScientific) temperature control. We analyzed [G1] in deuterated water (10 mg·mL^–1^) in the presence and absence of NaCl (10 mg·mL^–1^) (Table S1). To obtain dynamic viscosities, we multiplied the kinematic viscosity with the gravimetrically determined density of the sample.

To include sample viscosities in the calculation of hydrodynamic radii using the Stokes–Einstein relation, we estimate viscosities of deuterated water in varying salt concentrations by leveraging the known functional relation of the sample viscosity of nondeuterated water. The graphical plot of the dynamic viscosity of nondeuterated water at different NaCl concentrations is shown in Figure S1.^40^ To calculate analogue sample viscosities from deuterated water, we assumed the same relative changes and by starting from 0.001098 Pa·s for pure deuterated water at a temperature of 298.15 K (Table S1).

### Sample Preparation for In Situ Desalting

To investigate the utility of [G1] for native MS of soluble proteins in the presence of NaCl, protein samples were dissolved in 200 mM ammonium acetate (pH 7.0) and directly analyzed by native MS either without detergent or after adjusting the [G1] concentration in the sample to a final concentration of 0.04% (w/v) (Figure 1). Proteins were assayed at a final concentration of 10 μM, with the exception of myoglobin, which was analyzed at a concentration of 5 μM. For stepwise NaCl addition experiments, protein samples were dialyzed overnight at 4 °C against 200 mM ammonium acetate (pH 7.0) to ensure complete desalting. Dialyzed samples were then analyzed by native MS following the addition of NaCl in a 2-fold dilution series (0, 6.25, 12.5, 25 mM), either in the absence of detergent or after adjusting the [G1] concentration in samples to 0.04% (w/v). At 50 mM NaCl, the addition of [G1] did not enable acquisition of native MS data suitable for meaningful interpretation. Analogous BSA experiments were performed using tetraethylene glycol monooctyl ether (C_8_E_4_) and *n*-dodecyl-β-D-maltoside (DDM) at final detergent concentrations corresponding to approximately 2 × critical micelle concentration (cmc), namely 0.5% and 0.02% (w/v), respectively.

**Figure 1:**
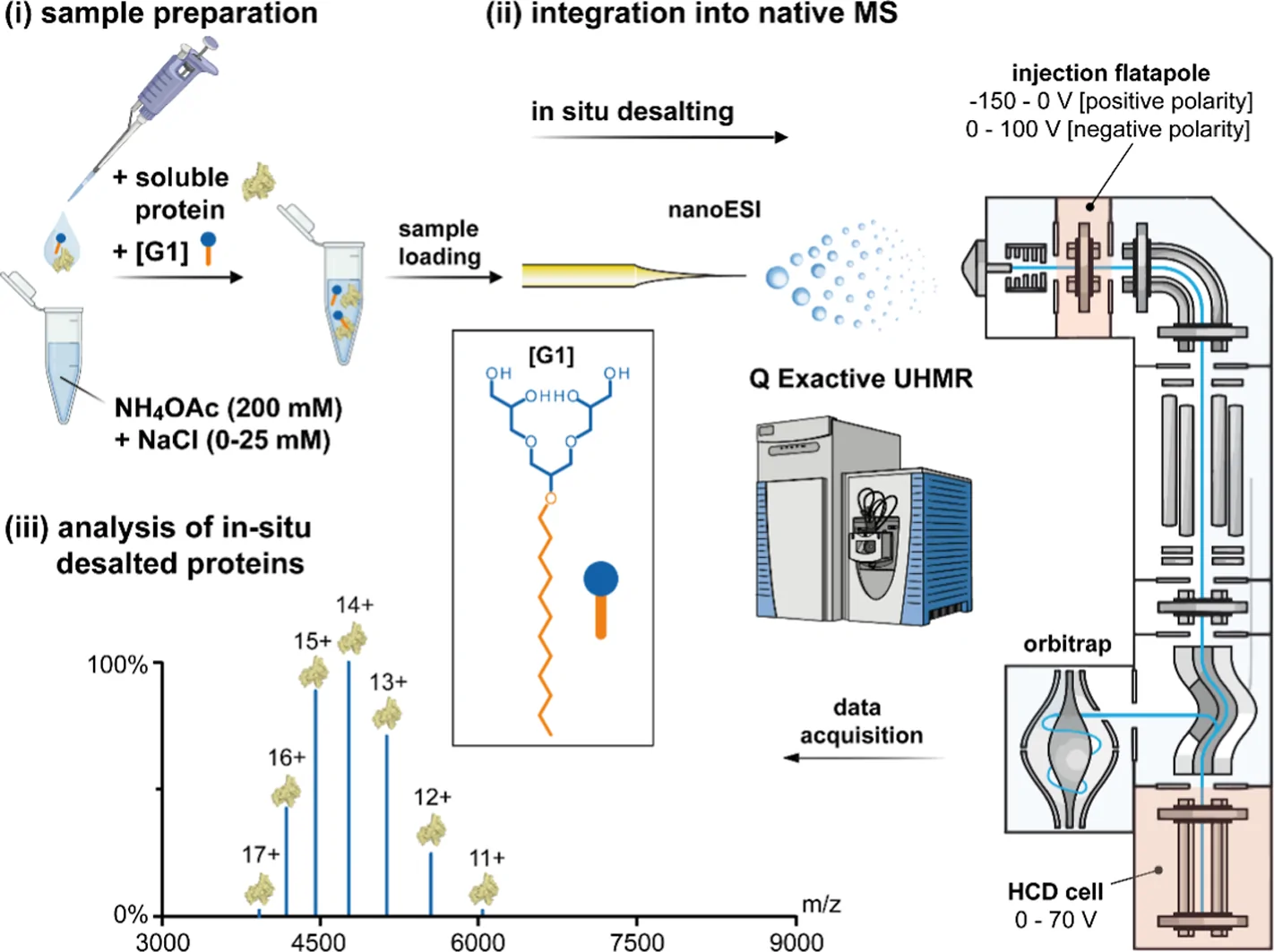
In situ desalting of soluble proteins in native MS enabled by [G1]. Schematic visualization of the practical integration of [G1] for native MS of in situ desalted proteins.

### Mass Spectrometry

To define the utility of [G1] for native MS of soluble proteins in the presence of NaCl, protein samples were directly introduced into the mass spectrometer using gold-coated capillary needles prepared in-house (Figure 1),^12^ with pulling parameters ensuring an inner tip diameter of ∼2 μm.^10,41,42^ MS analysis of [G1] was performed using the same nanoelectrospray setup. All experiments were carried out on a Q Exactive UHMR Hybrid Quadrupole-Orbitrap mass spectrometer (Thermo Fisher). The instrument was calibrated using a 10 mg·mL^–1^ solution of cesium iodide in water. For all experiments, the source temperature was maintained at 100 °C and the noise level was set to 3 rather than the default value of 4.64.

For native MS of soluble proteins, spectra were acquired in positive polarity for myoglobin, BSA, pyruvate Kinase, ADH, and in negative polarity for LptH. The instrument parameters used for MS spectra collection were the following: capillary voltage 1.1 kV (positive polarity) or 0.9 kV (negative polarity), scan range from 1000 to 20,000 *m*/*z*, HCD collision energy 0 (myoglobin, LptH, pyruvate kinase, ADH) or 70 V (BSA), source fragmentation 0 V, in-source trapping 0 V (myoglobin and BSA), −150 V (pyruvate kinase and ADH) or +100 V (LptH) (Figure 1 and Table S2). The ion transfer optics were set as follows: injection flatapole 5 V, interflatapole lens 4 V, bent flatapole 2 V, transfer multipole 0 V in positive polarity, while opposite values were set in negative polarity. The resolution of the instrument was 17,500 at *m*/*z* = 200 (transient time of 64 ms), nitrogen pressure in the HCD cell was maintained at approximately at 4×10^–10^ mbar.

For the MS analysis of [G1], a 0.04% w/v solution of the detergent in 200 mM ammonium acetate (pH 7.0) was introduced into the mass spectrometer in the absence or in the presence of NaCl (0.1, 0.2, 0.4, 0.8, 1.6 mg·mL^–1^). Spectra were acquired in both positive and negative polarity. The instrument parameters used for MS spectra collection were the following: capillary voltage 1.1 kV (positive polarity) or 0.9 kV (negative polarity), scan range from 350 to 2000 *m*/*z*, HCD collision energy 0, source fragmentation 0 V, in-source trapping 0 V. The ion transfer optics were set as follows: injection flatapole 5 V, interflatapole lens 4 V, bent flatapole 2 V, transfer multipole 0 V in positive polarity, while opposite values were set in negative polarity. The resolution of the instrument was 280,000 at *m*/*z* = 200 (transient time of 64 ms), nitrogen pressure in the HCD cell was maintained at approximately at 3.5×10^–9^ mbar.

Data were analyzed using the Xcalibur 3.0 (Thermo Scientific), NaViA,^43^ and UniDec^44^ software packages. Experiments were performed in triplicate (*n* = 3). Exact masses of proteins obtained under different conditions are summarized in Table S3. The potassium adduct of [G1], i.e., [M + K]^+^ represented the most abundant species under our conditions prior to the addition of NaCl, reflecting an intrinsic binding preference in the presence of ubiquitous background salt levels.

### Signal-to-Noise Ratio (S/N) Determination

To quantify the impact of [G1] on improvements in spectral quality, S/N was calculated directly from the raw *m*/*z* spectra exported as two-column text files (*m*/*z*, intensity) without additional smoothing or baseline subtraction. For each protein, a single representative, well-resolved charge-state peak (*z*) was selected for quantification and used consistently across matched comparisons. The signal intensity (*S*) was defined as the maximum peak apex intensity of the selected charge state in the raw *m*/*z* spectrum. The baseline (B) and noise (σ) were determined from a peak-free noise window on the lower-*m*/*z* side of the selected charge state, chosen to be close to the analyte peak (typically on the order of ∼100 *m*/*z* lower when feasible) but positioned to avoid any analyte, adduct, or solvent-cluster features. The baseline was taken as the median intensity in the noise window and σ as the standard deviation of intensities in the same window. S/N was calculated as

$$S/N=\frac{S-B}{\sigma }$$

Noise-window placement and width were held constant within each protein and NaCl condition when comparing data sets in the absence and in the presence of [G1], ensuring that differences in S/N reflect changes in spectral quality rather than changes in evaluation parameters.

### Lipid and Drug Binding Experiments

To monitor the impact of NaCl in ligand binding, 1-palmitoyl-2-oleoyl-*sn*-glycero-3-phospho-1′-rac-glycerol (16:0/18:1 PG, POPG) was purchased from Avanti Polar Lipids as a powder and stock solutions were prepared following a previously established method.^45^ Semaglutide was purchased from Selleck Chemicals as a powder. POPG and semaglutide were diluted in 200 mM ammonium acetate (pH 7.0) containing [G1] at a concentration of 0.04% (w/v). BSA-POPG binding experiments were performed in analogy to a previous procedure^46^ by diluting POPG in 200 mM ammonium acetate (pH 7.0) supplemented with [G1] 0.04% (w/v) and adding it to BSA (10 μM final) at increasing POPG concentrations (15, 30, and 60 μM). BSA-semaglutide binding experiments were performed using an analogous protocol, with semaglutide added to BSA at the same concentrations under the same buffer and [G1] conditions. Experiments were conducted in 200 mM ammonium acetate (pH 7.0) in the absence of NaCl or with 3.125 or 6.25 mM NaCl reflecting physiologically relevant protein-NaCl stoichiometries relative to our biophysically relevant protein concentration.^47–50^ The complexity of protein–ligand mixtures did not enable the acquisition of native MS data suitable for meaningful interpretation for NaCl concentrations above 6.25 mM.

Relative intensities of apo and ligand-bound protein species were calculated from the native MS data acquired at each ligand concentration and NaCl concentration. For each spectrum, peak intensity values corresponding to the apo protein and to all detectable ligand-bound protein species were extracted from the deconvoluted data using UniDec. The total apo intensity was calculated considering all peaks assigned to the unbound protein, whereas the total ligand-bound intensity included all peaks assigned to protein–ligand complexes. The relative intensity of ligand-bound protein was then determined by dividing the summed intensity of ligand-bound species by the total protein intensity, defined as the sum of apo and ligand-bound intensities. These values were calculated independently for every spectrum obtained under each NaCl condition. Relative intensities from three independent replicates (*n* = 3) were averaged and plotted against ligand concentration, with error bars representing the standard deviation (±SD).

## Results and Discussion

### [G1] Binds NaCl in Solution

Investigating if [G1] interacts with NaCl in solution is of pivotal importance for understanding the role of the detergent in desalting. Supercharging in situ desalting reagents bind sodium ions in solution, which reduces sodium adduct formation in the gas phase.^21,22^ To test whether [G1] binds sodium ions in solutions, we compared the chemical shifts of the detergent protons in ^1^H NMR, before and after adding NaCl. In ^1^H NMR experiments, all proton signals from the detergent were shifted to lower ppm values after adding NaCl (Figure 2). This shift was less pronounced for the triglycerol backbone (∼7 Hz) than for the aliphatic tail (∼8 Hz) (Figure 2). This observation is characteristic for polyether/polyol structures and can be explained by interactions of the detergent headgroup with ions.^51^ Chloride anions likely associate with the first hydration layer of the triglycerol headgroup, whereas sodium cations remain more strongly hydrated and interact less specifically with polyether/polyols.^51^ The relatively hydrophobic characters of chloride ions and triglycerol, compared to hydrated sodium ions and water, favor this association.^51^ The hydrated sodium ions, however, cause a stronger upfield shift of the water (16.9 Hz) than for detergent signals (7–8 Hz). Furthermore, the electron-withdrawing nature of chloride is expected to reduce the shift of the signals of the triglycerol headgroup to lower ppm values compared to the alkyl tail, consistent with our ^1^H NMR data.^51^ Our NMR experiments indicate the headgroup of [G1] interacts with NaCl.

**Figure 2:**
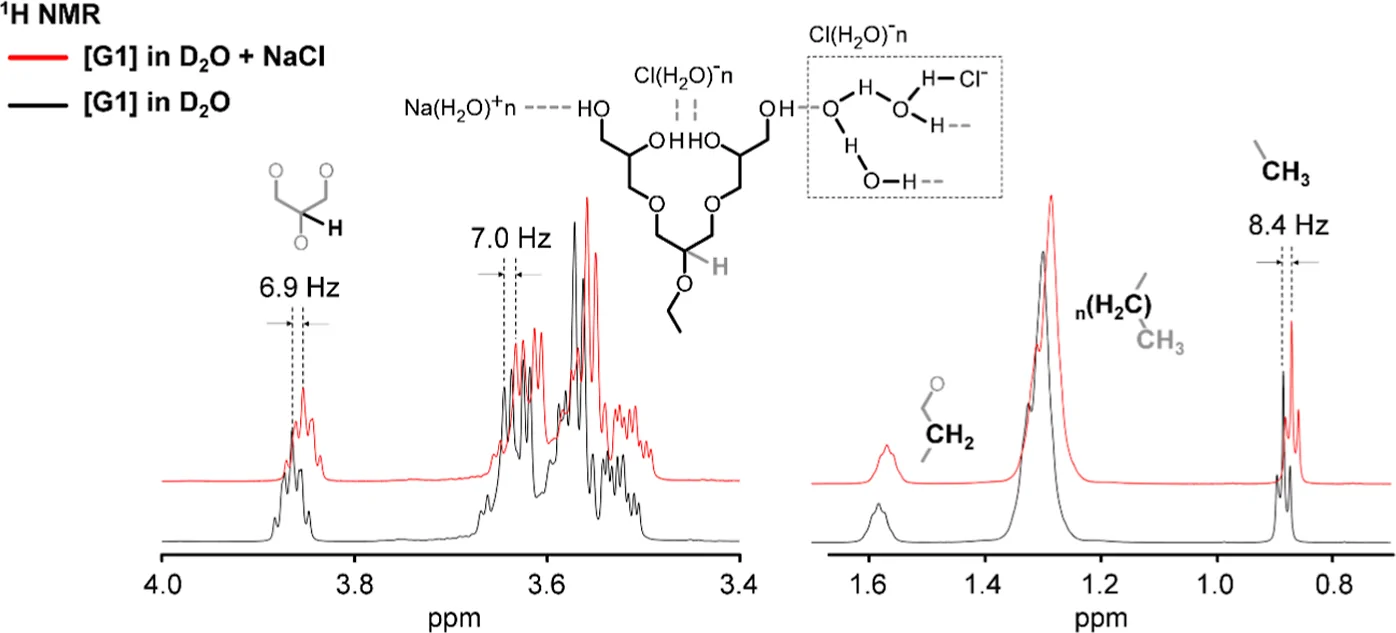
^1^H NMR spectra of [G1] in D_2_O in the absence (black) and presence (red) of NaCl. Signal assignments and proposed [G1]-NaCl interactions are depicted by bold black labels and lines. Representative chemical shift changes are reported in Hertz (Hz).

^1^H DOSY measurements support the hypothesis that [G1] interacts with NaCl, as evidenced by a reduced detergent diffusion coefficient (Figure S2). Similar changes were observed in ^23^Na DOSY before and after detergent addition (Figure S3). To assess whether binding of NaCl to [G1] varies with concentration, we analyzed ^1^H DOSY data across a range of NaCl concentrations. Increasing the concentration of NaCl led to smaller detergent diffusion coefficients and larger hydrodynamic radii (Table S1). To exclude viscosity effects, we calculated viscosity-corrected hydrodynamic radii, which again confirmed that the hydrodynamic radii of [G1] increased with increasing NaCl concentration (Table S1). [G1] interacts with NaCl in solution in a concentration-dependent manner, suggesting potential as a desalting reagent in native MS of soluble proteins.

### [G1] Enables In Situ Desalting of Soluble Proteins in Native MS

To test whether adding [G1] to soluble proteins delivers an in situ desalting strategy for native MS, holomyoglobin was analyzed as a lyophilized powder that was solublized in ammonium acetate solution with and without [G1] (Figure 3a–c) (Table S3). The commercial holomyoglobin powder contained salt. Following native MS without detergent, we observed mass spectra of holomyoglobin with up to 8 sodium adducts (Figure 3c). In the presence of [G1], we observed only sodium-free holomyoglobin and the overall spectral quality increased, including a 12-fold improvement in S/N (Figure 3d) (Table S4). Dissolving a salt-containing protein in ammonium acetate solution along with [G1] enabled the facile native MS analysis of the sodium-free holo form of myoglobin. High-quality spectra were obtained without the need for denoising, despite conditions that are typically associated with increased spectral complexity that commonly require strategies to improve S/N values, such as higher collisional activation^52^ or post–acquisition processing.^53^

**Figure 3:**
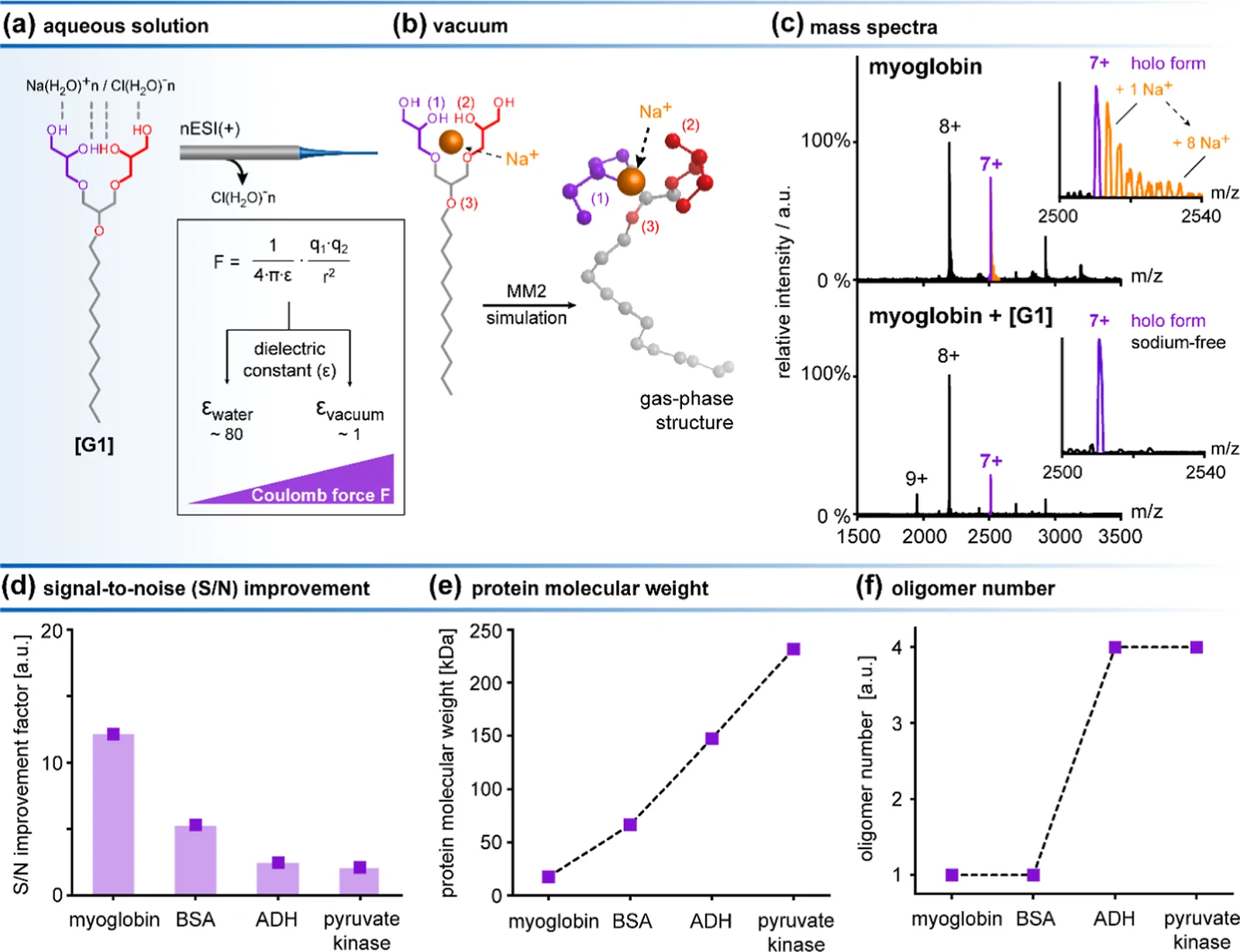
[G1] enables desalting native MS of myoglobin. (a) Possible interactions between the [G1] headgroup and ions of NaCl in aqueous solution. (b) Proposed binding of the [G1] headgroup to sodium ions in vacuum. Positive electrospray ionization preferentially transmits sodium over chloride ions. Hydrogen atoms are omitted from the gas-phase structure of [G1] to better visualize the proposed sodium binding site. (c) Native mass spectra of commercial myoglobin dissolved in 200 mM aqueous ammonium acetate (top) and after addition of 0.0.04% (w/v) (0.9 mM) [G1] (bottom). (d) Summary of signal-to-noise improvement factors, (e) protein molecular weights, and (f) oligomer numbers of commercial protein samples sprayed with [G1] compared to detergent-free samples.

The removal of sodium cations from holomyoglobin by [G1] can be rationalized by considering the behavior of ions during the electrospray ionization process. In positive polarity, sodium ions are separated from chloride ions and transmitted together with proteins and detergents (Figure 3a). During desolvation, the dielectric constant of the analyte environment decreases approximately by factor 80, causing electrostatic interactions to increase (Figure 3a).^54^ We propose the sum of these environmental changes forces [G1] to bind sodium cations during electrospray ionization and desolvation (Figure 3b).

[G1] binds NaCl in a concentration-dependent manner in solution. To test whether this behavior persists during electrospray ionization, we monitored the relative intensity of sodium adducts formed with [G1] across varying NaCl concentrations in solution. The relative intensity of sodiated [G1] increased with increasing NaCl concentration (Figure S4). Molecular dynamics simulations suggest oxygen atoms of triglycerol headgroups chelate sodium ions in vacuum (Figure 3a).^23,55^ Prior tandem MS studies of sodiated β-lactoglobulin complexes with [G1] report that the detergent strips sodium cations away from protein surfaces upon collision-induced dissociation.^23^ Although our methodology does not allow further clarification of the desalting mechanism, [G1] likely competes with proteins for sodium adduction during electrospray ionization, with additional sodium removal potentially occurring upon dissociation from protein-detergent complexes.

To test whether the improvements in S/N extend to other proteins, we analyzed BSA, ADH, and tetrameric pyruvate kinase. The relative improvement in S/N decreased with increasing protein molecular weight and oligomer number (Figure 3d–f) (Table S4). To enable native MS of ADH and pyruvate kinase, ion transmission had to be improved by increasing the in-source trapping voltage from 0 to 150 V. Similar to myoglobin, this led to increased relative intensities of apo states of ADH and pyruvate kinase (Figure S5). In contrast to myoglobin, we did not observe sodium adducts for both proteins, suggesting collisional activation, due to in-source trapping, led to desalting.^56^ Nevertheless, [G1] again improved spectral quality, as reflected by elevated S/N improvement factors for the higher-molecular-weight proteins (Figure 3d). Our findings indicate [G1] can enhance spectral quality through a mechanism beyond the mitigation of sodium adduct formation. Solvent properties, particularly surface tension, can significantly influence protein charging and signal intensity in native MS, with lower surface tension favoring the stability of the electrospray process leading to more efficient ion formation.^57–60^ Nonionic detergents and other surfactants are well-known to reduce surface tension as they accumulate at the liquid–air interface.^61^ The increase in S/N improvement factors obtained upon adding [G1] to protein samples are in line with an improvement in electrospray efficiency arising from a reduction in surface tension, alongside competition with proteins for sodium adduction in electrospray ionization (Figure 3d) (Figure S5) (Table S4).^23,55,62^

Moreover, the use of [G1] in our method did not lead to an appreciable shift toward higher protein charge states, with charge-state distributions remaining broadly comparable in the presence and absence of [G1] (Figures 3a and S5 to S8). [G1] enables desalting and improves signal quality without supercharging protein analytes away from the native regime. In native MS, preservation of compact, natively folded proteins and assemblies limits charging during electrospray ionization, resulting in lower charge states and narrower charge-state distributions compared with denaturing conditions, where unfolded proteins expose more ionizable sites. Systematic shifts to higher protein charge states are commonly associated with a destabilization of noncovalent interactions.^6,63–67^ Consistent with this framework, prior studies have shown that nonionic detergents can maintain or even reduce protein charge states while supporting the preservation of noncovalent interactions in native MS.^23,26,55,68,69^ This contrasts with classical supercharging additives, e.g., *m*-nitrobenzyl alcohol, which increase protein charge states and destabilize protein complexes.^61,70^ Overall, these observations further support [G1] as an effective in situ desalting reagent, enabling native MS of soluble protein complexes with soft activation conditions.

### [G1] Increases NaCl Tolerance in Native MS of Soluble Proteins

A key advantage of [G1] is the ease of its implementation that can simplify the analysis of biophysically relevant oligomeric states. BSA is the bovine analogue of human serum albumin, a protein that is widely studied in context with pharmaceutical formulations and biochemical preparations,^71^ but is prone to aggregate.^72^ Direct infusion of BSA from aqueous ammonium acetate solution into our mass spectrometer led us to the obtainment of monomers, dimers, and trimers (Figure 4a).^73^ After adding [G1], we obtained exclusively monomeric BSA (Figure 4a). Protein aggregation is a significant source of unspecific bioactivity,^74^ which can be reversed by the addition of detergent.^75,76^ Control experiments with size-exclusion chromatography indicate that dissolving BSA in [G1]-containing buffer did not noticeably shift the size distribution profile of protein particles in solution (Figure S9).

**Figure 4:**
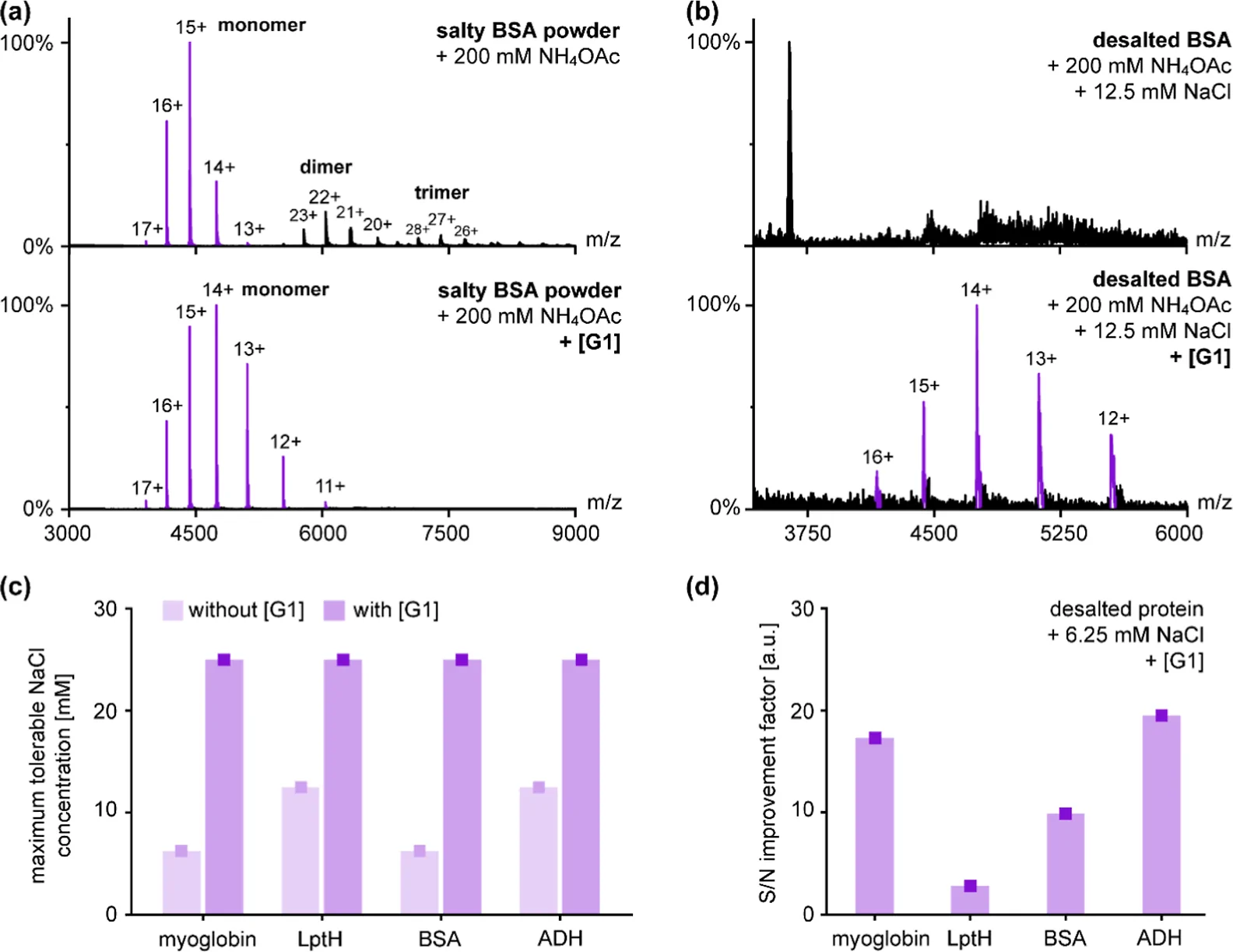
[G1] improves S/N and tolerable NaCl concentration in native MS. (a) Mass spectra obtained from salty BSA powder dissolved in ammonium acetate with and without detergent. (b) Mass spectra obtained from desalted BSA upon controlled addition of NaCl with and without detergent. (c) Bar chart showing highest tolerable NaCl concentration that enabled interpretable protein mass spectra without and with [G1] under assay conditions. (d) Bar chart showing S/N improvement factors obtained from desalted proteins analyzed after controlled addition of NaCl and [G1] compared to detergent-free control.

This indicates that [G1] can decouple the solution-state oligomer distribution of BSA from the one observable in native MS, providing an in situ desalting tool that simplifies spectra and the analysis of biophysically relevant oligomeric states.

Another advantage of [G1] is its ability to increase the maximum concentration of NaCl that enables interpretable mass spectra. To identify the maximum concentration of NaCl tolerated in our method, we desalted our proteins and repeated native MS upon adding NaCl in 2-fold dilution steps. [G1] consistently increased the maximum tolerable NaCl concentration in our method to 25 mM, irrespective of the protein (Figure 4c). Furthermore, [G1] led to improved S/N values at a comparable NaCl concentration across our proteins (Figure 4d) (Table S4). Overall, we were unable to obtain interpretable native MS data in the presence of >12.5 mM NaCl without adding [G1] (Figures 4b, S5–S8). [G1] increased the amount of NaCl tolerated in native MS of soluble proteins, enabling robust measurements under millimolar NaCl concentrations while maintaining spectral resolution with improved S/N.

### Lipid and Drug Binding Enabled by [G1]

Techniques enabling the analysis of lipid and drug binding to soluble proteins can help with characterizing biomolecular interactions.^77^ In native MS, detergent micelles serve as vehicles that increase solubility and mediate binding of proteins to hydrophobic ligands.^46^ To monitor the binding of lipids and hydrophobic drugs to soluble proteins, detergents must be used above a critical concentration at which micelles are formed (cmc).^46^ Detergents with low cmc values, like C_10_ × 10^6^ (cmc ∼0.9 mM), have been used at 10x cmc to enable the analysis of lipid binding to soluble proteins.^46^ Lower detergent concentrations have not led to the formation of protein–lipid complexes.^46^ Interestingly, this detergent concentration is 9 times higher than the utilized concentration of [G1] in our assay (∼1 mM). As exemplified in the case of BSA, the use of [G1] can enable a more efficient analysis of BSA-POPG lipid binding by native MS (Figure 5a).^46^

**Figure 5:**
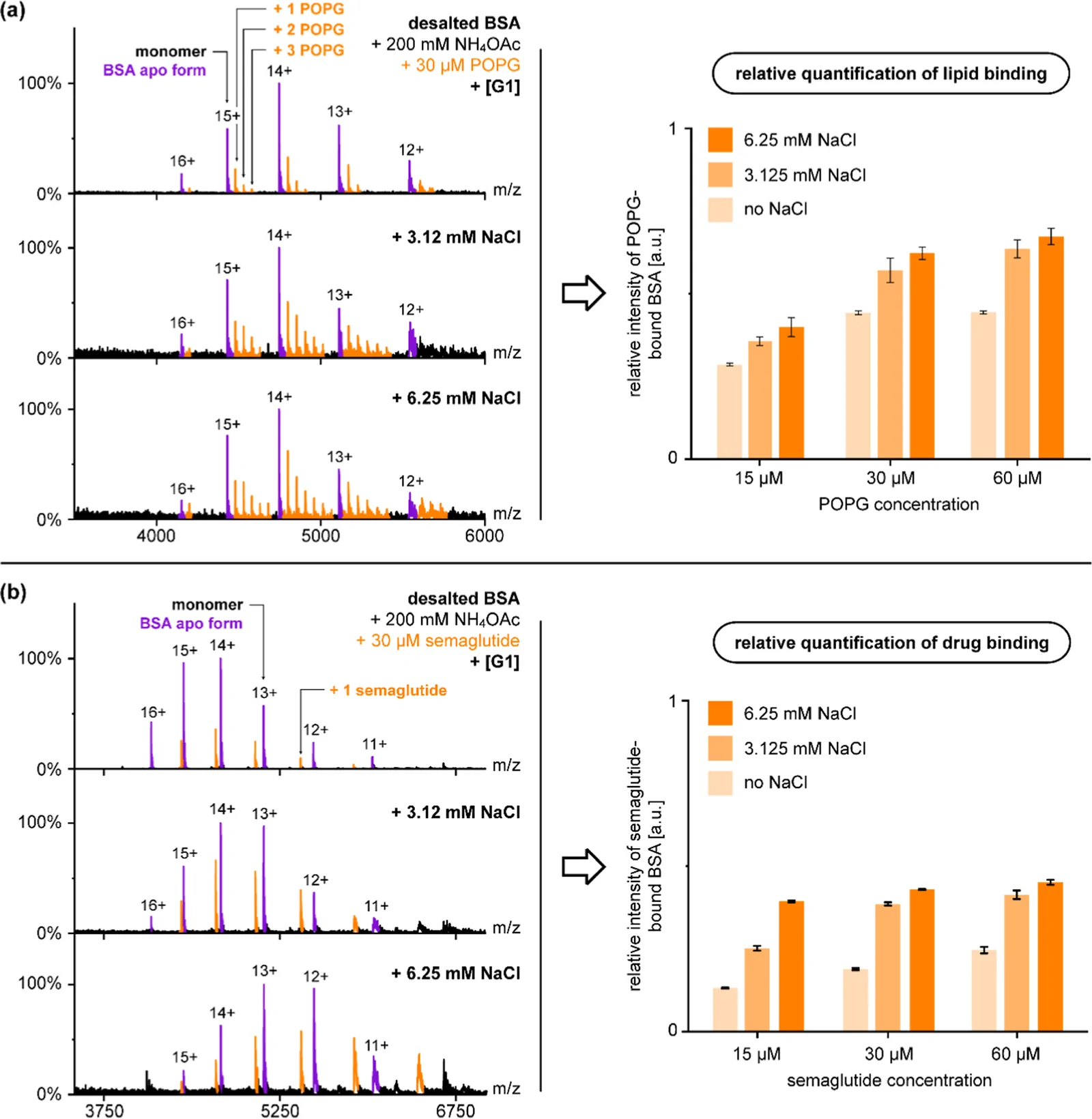
[G1] enables native MS of lipid and drug binding in the presence of NaCl. Mass spectra of BSA in the presence of (a) POPG lipid or (b) semaglutide drug at varying NaCl concentrations. Bar charts visualize relative intensities of ligand-bound BSA fractions at different NaCl concentrations. Data are shown with standard deviation (±SD) from three independent repeats (*n* = 3).

Lipid binding has previously been enabled by nonionic detergents, including C_8_E_4_ and DDM, which are widely used in native MS.^78^ In direct comparison, however, [G1] endowed our method with a substantially greater salt tolerance. While C_8_E_4_ and DDM permitted clear detection of BSA up to 6.25 mM NaCl, with poorly resolved spectra at 12.5 mM (Figure S10), [G1] enabled detection of BSA at NaCl concentrations up to 25 mM (Figure 4c). NMR data from Zhu et al.,^51^ together with our findings, suggest that interactions between polyether/polyol detergents and NaCl commonly occur in solution. The superior salt tolerance in our method enabled by [G1] is likely due to a better ability to mitigate protein-salt adduct formation in electrospray ionization compared to C_8_E_4_ and DDM.

Previous studies on membrane protein–ligand interactions highlighted the utility of integrating [G1] and native MS for monitoring the impact of NaCl on agonist binding to a GPCR.^28^ To test whether [G1] can be used to study the role of NaCl on lipid binding to soluble proteins, we monitored relative intensities of BSA and its complexes with POPG lipid at different NaCl concentrations from standard nanoelectrospray emitters (Figure 5a). BSA has a well-known propensity to bind POPG,^46^ in line with its biological role as a carrier for amphiphilic molecules.^79^ Interestingly, the relative intensity of BSA-POPG complexes increased with increasing NaCl concentration (Figure 5a). Our findings indicate that adding NaCl to ammonium acetate solution stabilizes POPG binding to BSA.

To test whether the ability to analyze the regulatory impact of NaCl on biomolecular interactions extends to drug binding, we monitored relative intensities of BSA and its complexes formed with semaglutide, a therapeutic glucagon-like peptide-1 receptor agonist whose prolonged plasma half-life relies on albumin binding mediated by its C18 fatty diacid side chain.^80^ Again, the relative intensity of BSA-semaglutide complexes increased with increasing NaCl concentration (Figure 5b). Adding NaCl to ammonium acetate solutions stabilizes drug binding to BSA. The use of [G1] in native MS has already proven powerful for dissecting membrane protein–lipid interactions and salt-dependent ligand binding to GPCRs.^24,26–29,33,81^ Our findings define the utility of [G1] for a native MS workflow that uncovers changes in relative intensities of soluble protein complexes with lipids and drugs under defined NaCl concentrations.

The influence of NaCl on hydrophobic interactions between BSA and amphiphilic interfaces has been extensively investigated. For example, increasing NaCl concentrations have been shown to enhance BSA adsorption to sodium dodecyl sulfate, lecithin films, and block copolymer micelles.^82–84^ Methods that enable a systematic analysis of salt-driven effects on biomolecular interactions are valuable for guiding the rational design of hybrid materials. A major challenge in such analyses is the intrinsic heterogeneity of supramolecular systems. Condensed-phase techniques average the results obtained from the entire population of species within a sample.^55^ In solution, proteins and their complexes with ligands are in dynamic equilibrium, rendering the unambiguous analysis of individual complexes difficult. Combining [G1] with native MS extends the repertoire of methods to work around this challenge by enabling the relative quantification of individual protein–ligand complexes in the presence of many others and NaCl, including lipids and drugs. Future studies examining the effects of additional salts and salt mixtures in the absence of ammonium acetate may further refine the model system and enhance its relevance to in vivo conditions.

## Conclusion

In summary, we established [G1] as a simple-to-apply additive that enables native MS of soluble protein complexes in the presence of NaCl. [G1] increases tolerable NaCl concentrations up to 25 mM while improving S/N and preserving biomolecular interactions and native charge states. By remaining compatible with standard nanoelectrospray emitters, [G1] enables native MS studies under conditions previously unattainable without this additive. NMR measurements indicate a concentration-dependent NaCl complexation by [G1] in solution, consistent with an improved spectral quality of desalted protein complexes observed by native MS. Compared to supercharging reagents,^70,85^ [G1] maintains native charge states while delivering efficient desalting. This facilitated native MS of soluble proteins from monomers to tetramers with molecular weights up to 231 kDa, suggesting broad applicability of our method beyond this work. Furthermore, our findings spotlight the utility of [G1] for monitoring NaCl-dependent stabilization of protein–ligand interactions, including binding to lipids and drugs. Compared with traditional offline desalting strategies, such as microdialysis, size-exclusion chromatography, or ultrafiltration,^7–9,11,13^ [G1] requires no additional sample handling and remains compatible with routine workflows. Taken together, our results establish [G1] as a versatile additive that extends the tolerable NaCl range in native MS of soluble proteins and enables controlled, biophysically informed investigations of how physiologically relevant ions reshape protein–ligand binding landscapes. Future studies examining additional salts, salt mixtures, and protein–ligand systems will refine this model and inform the rational design of next-generation desalting reagents. Our findings position [G1] as a practical, easy-to-apply additive that enhances salt tolerance and spectral quality in native MS, facilitating the biophysical analysis of challenging membrane proteins and soluble protein assemblies.

## Supplementary Material



## Notes

Additional supplementary figures and tables are provided that inform on dynamic viscosity of H_2_O in dependence of NaCl concentration; ^1^H DOSY spectra of [G1]; ^23^Na DOSY; relative MS intensities of sodiated [G1]; protein mass spectra of myoglobin, LptH, ADH, pyruvate kinase; theoretical and experimental masses of proteins; viscosity data determined by Ubbhelode experiments; diffusion coefficients and hydrodynamic radii of detergent in dependence of the NaCl concentration; all native MS data were uploaded to the ProteomeXchange Consortium via the PRIDE^86^ repository with the data set identifier PXD074233 (PDF)

## Abbreviations

[G1]: first-generation, dendritic triglycerol detergent
ADH: alcohol dehydrogenase
BSA: bovine serum albumin
C_8_E_4_: tetraethylene glycol monooctyl ether
cmc: critical micelle concentration
DDM: *n*-dodecyl-β-D-maltoside
GPCR: G protein-coupled receptor
LptH: lipopolysaccharide transport protein H
*m*/*z*: mass-to-charge ratio
NaCl: sodium chloride
native MS: native mass spectrometry
NMR: nuclear magnetic resonance
S/N: signal-to-noise ratio
SD: standard deviation
TEV: tobacco etch virus
