# Supplementary material for: Native Mass Spectrometry of Soluble Proteins with Sodium Chloride Enabled by a Dendritic Detergent
Source: ACS Meas Sci Au. 2026 Jun 9;6(4):1038–48. doi: 10.1021/acsmeasuresciau.6c00038 (PMC13495734; doi:10.1021/acsmeasuresciau.6c00038)
Supplement: Supplementary file 1 [file tg6c00038_si_001.pdf]

Supporting Information

Native Mass Spectrometry of Soluble Proteins with Sodium Chloride enabled by a Dendritic Detergent

Francesco Fiorentino*[1], Wolf Hiller*[2], Leonhard H. Urner*[2]

[1] Department of Biochemical Sciences, Sapienza University of Rome, Rome, Italy

[2] Department of Chemistry and Chemical Biology, TU Dortmund University, Dortmund, Germany

***Correspondence:**

Francesco Fiorentino (f.fiorentino@uniroma1.it);

Wolf Hiller (wolf.hiller@tu-dortmund.de);

Leonhard H. Urner (leonhard.urner@tu-dortmund.de).

Supplementary Figures

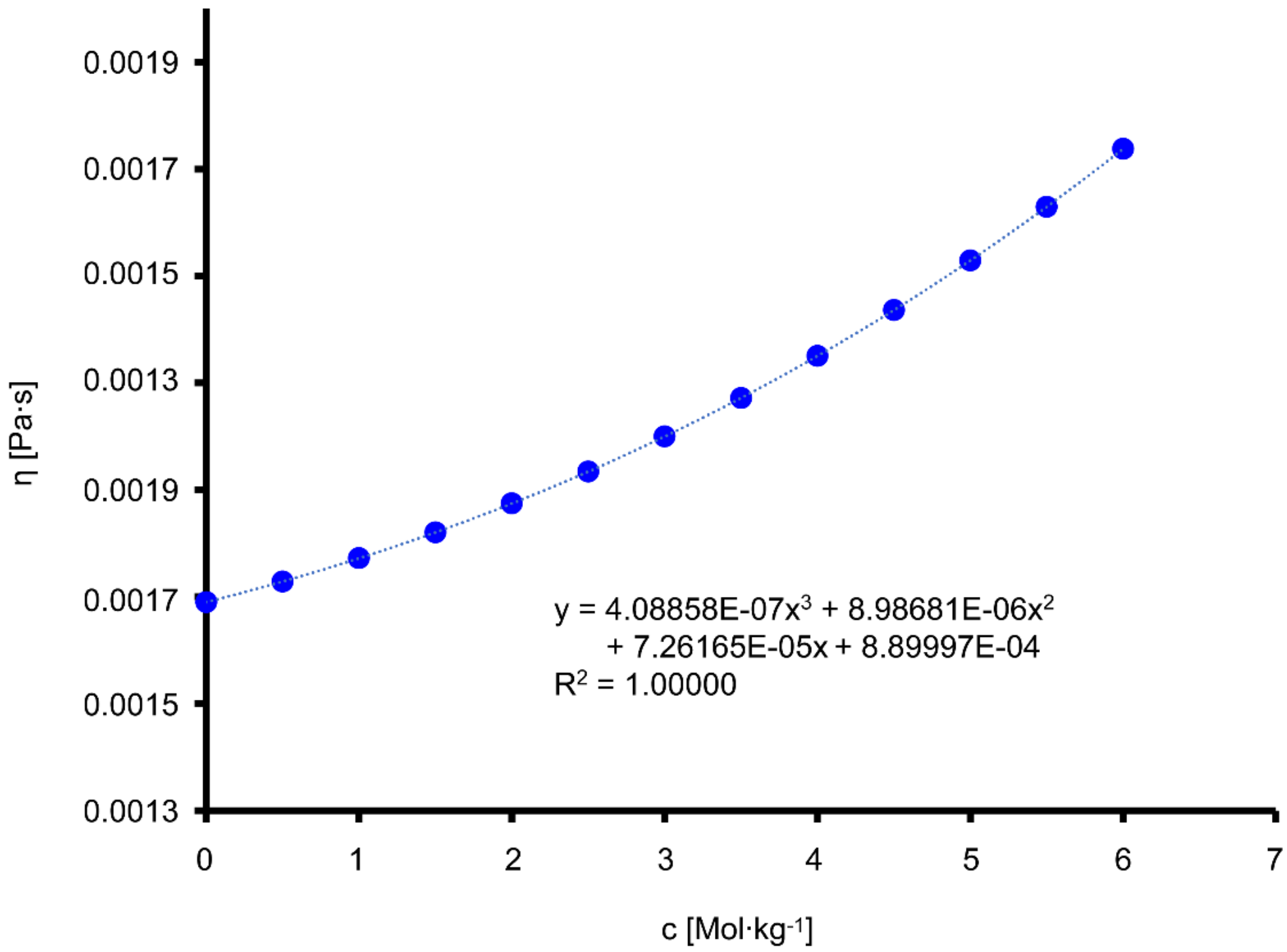

Figure S1. Dynamic viscosity of H_2O in dependence of sodium chloride concentration at 298.15 K (plotted from Kerstin and co-workers[41]).

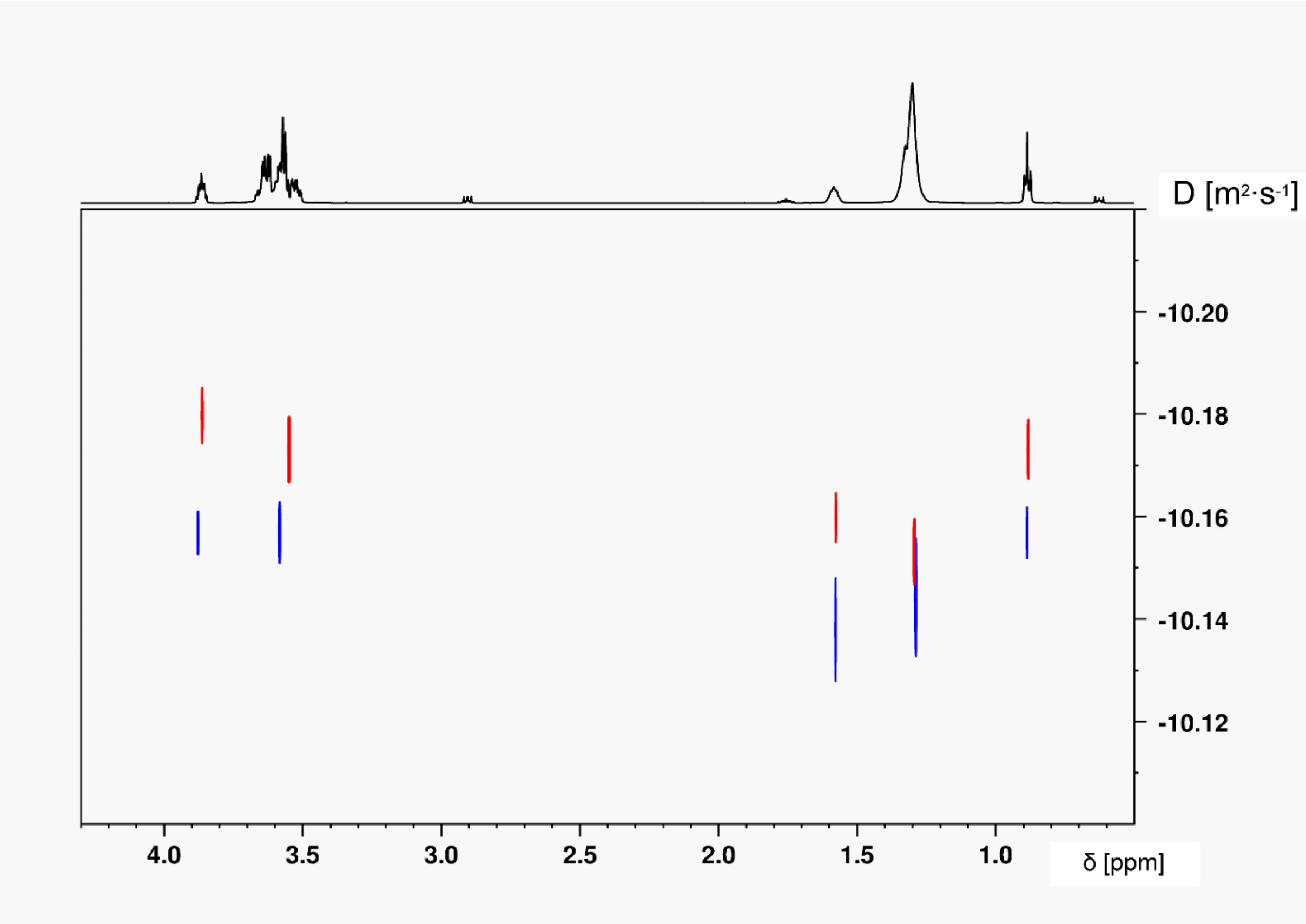

Figure S2. ^{1}H DOSY spectra of [G1] without sodium chloride (blue) and with 10 mg NaCl (red).

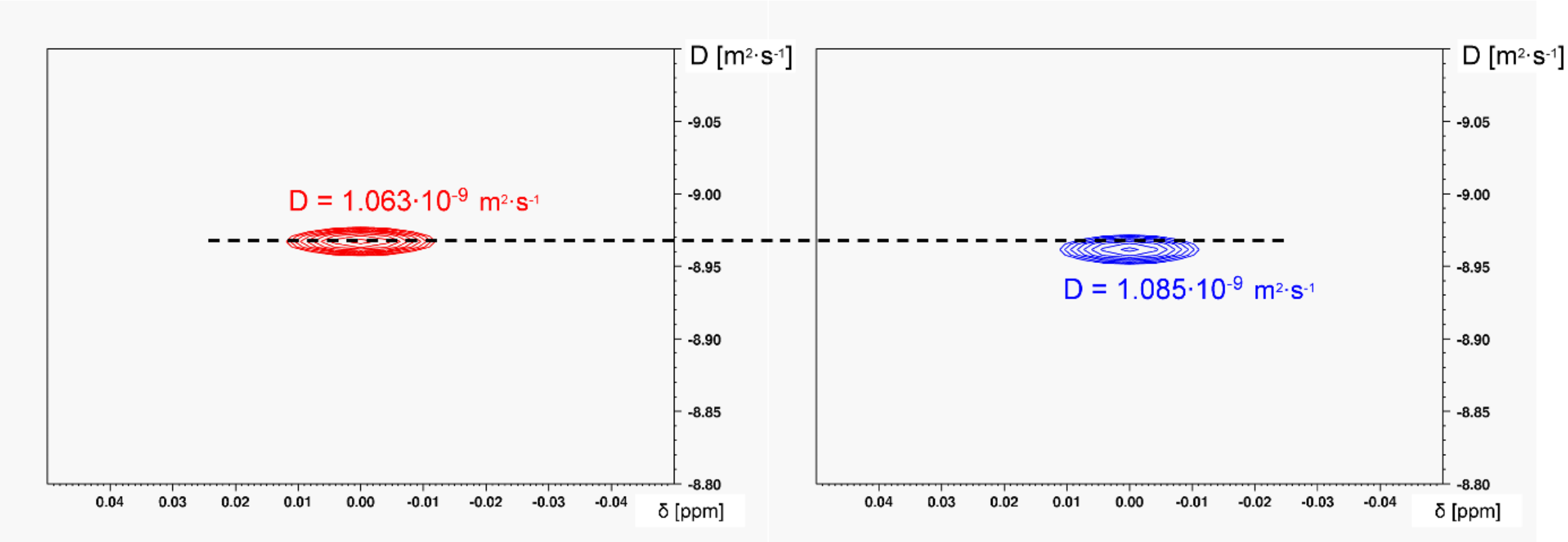

Figure S3. ^{23}Na DOSY of detergent (10 mg·ml^{-1}) with sodium chloride (10 mg·ml^{-1}) (left panel) and only sodium chloride (10 mg·ml^{-1}) in deuterated water (right panel).

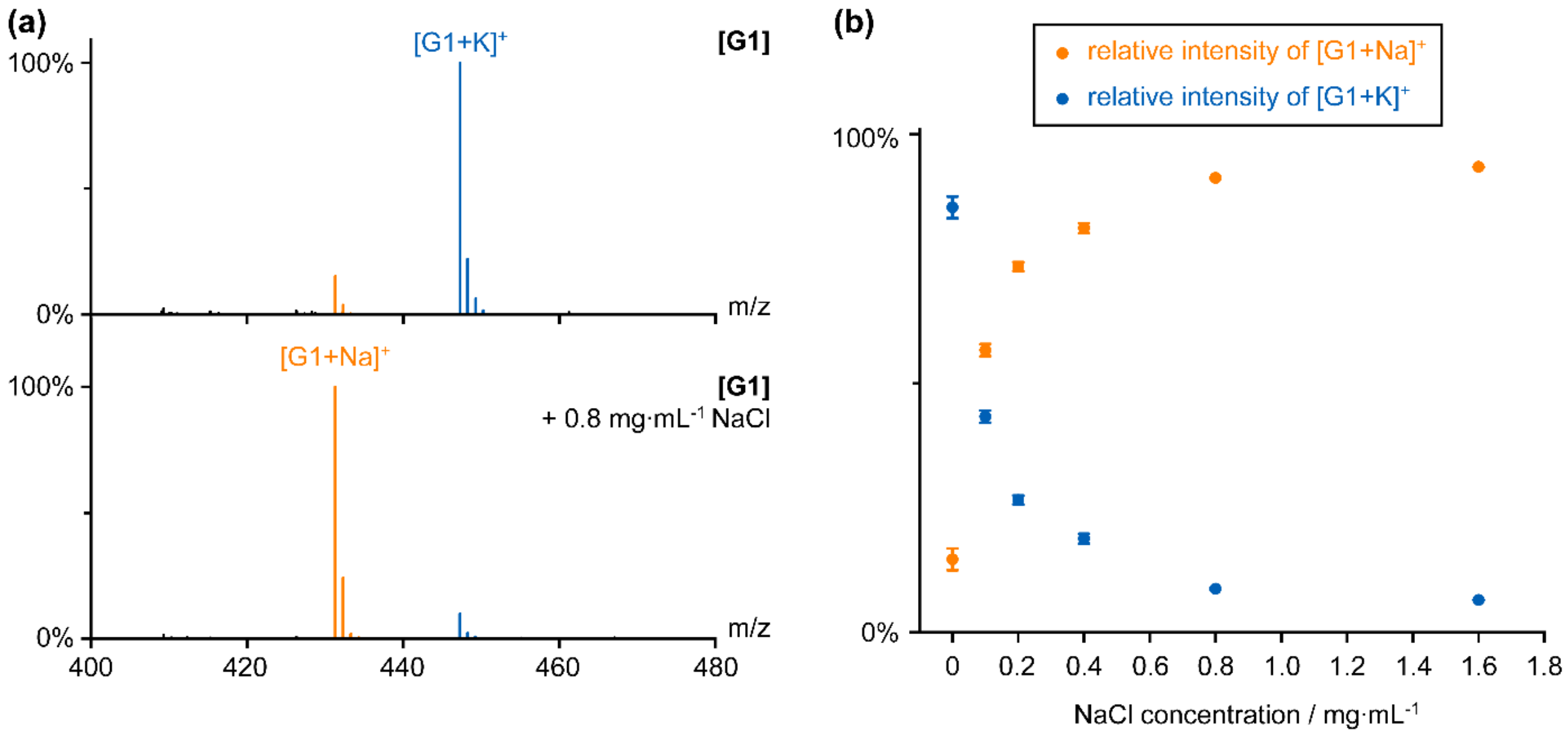

Figure S4. Impact of sodium chloride on relative intensity of sodiated [G1]. (a) Mass spectra of [G1] analyzed with and without sodium chloride in solution. (b) Diagram showing relative intensities of sodium and potassium adducts of [G1] against different sodium chloride concentrations in solution.

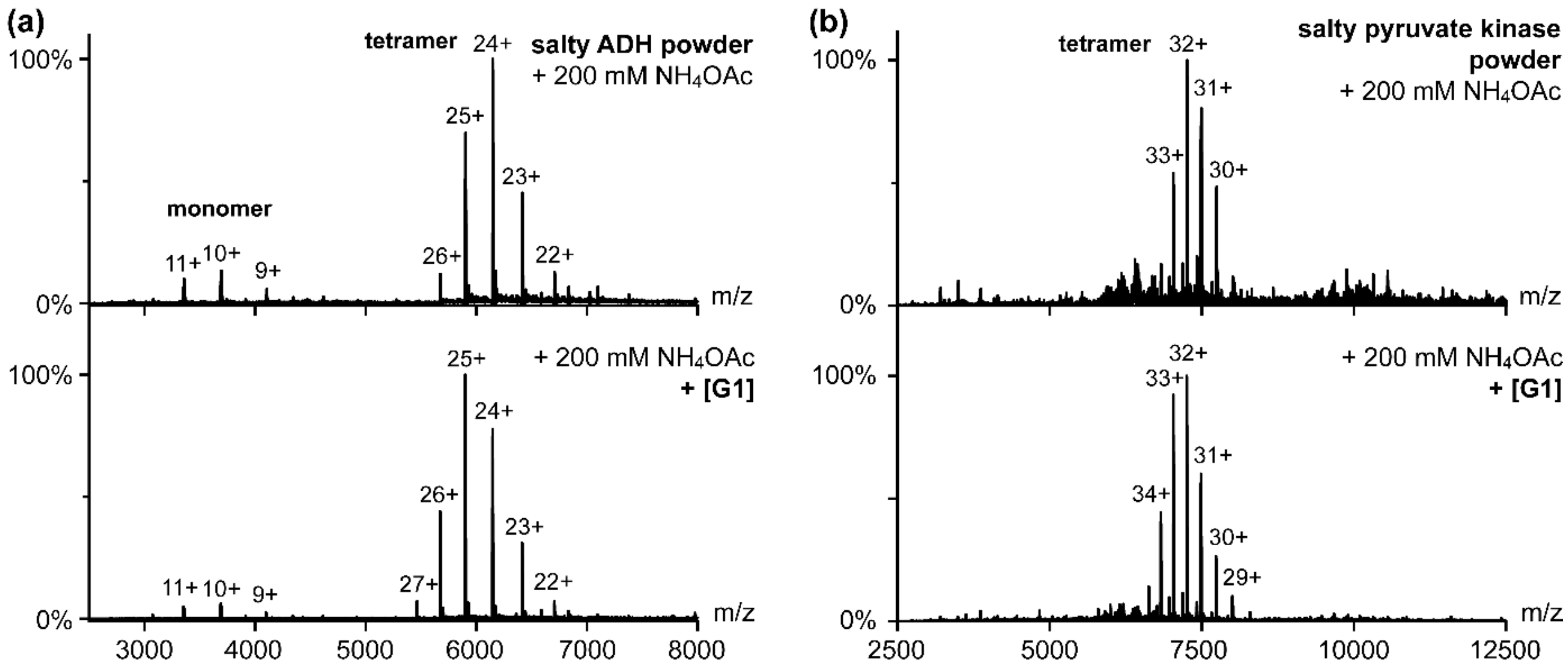

Figure S5. Impact of [G1] on native MS solubilized protein powders. (a) Mass spectra obtained from alcohol dehydrogenase powder (MW ~ 66 kDa) dissolved in ammonium acetate with and without detergent. (b) Mass spectra obtained from pyruvate kinase powder (MW ~ 231 kDa) dissolved in ammonium acetate with and without detergent.

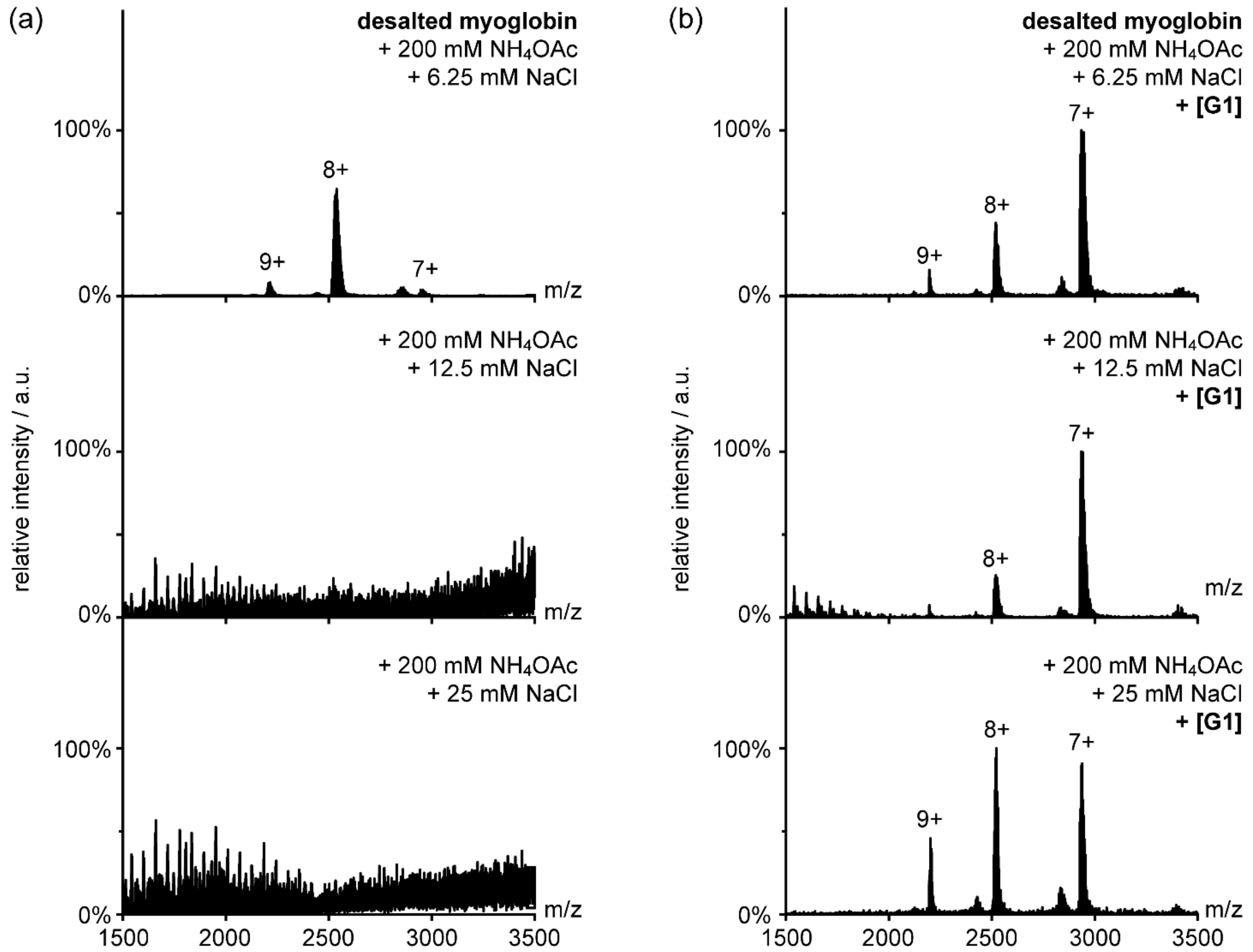

Figure S6. Sodium chloride tolerance in native MS of myoglobin with [G1]. (a) Mass spectra obtained from desalted myoglobin upon controlled addition of sodium chloride are compared with (b) mass spectra obtained upon addition of [G1].

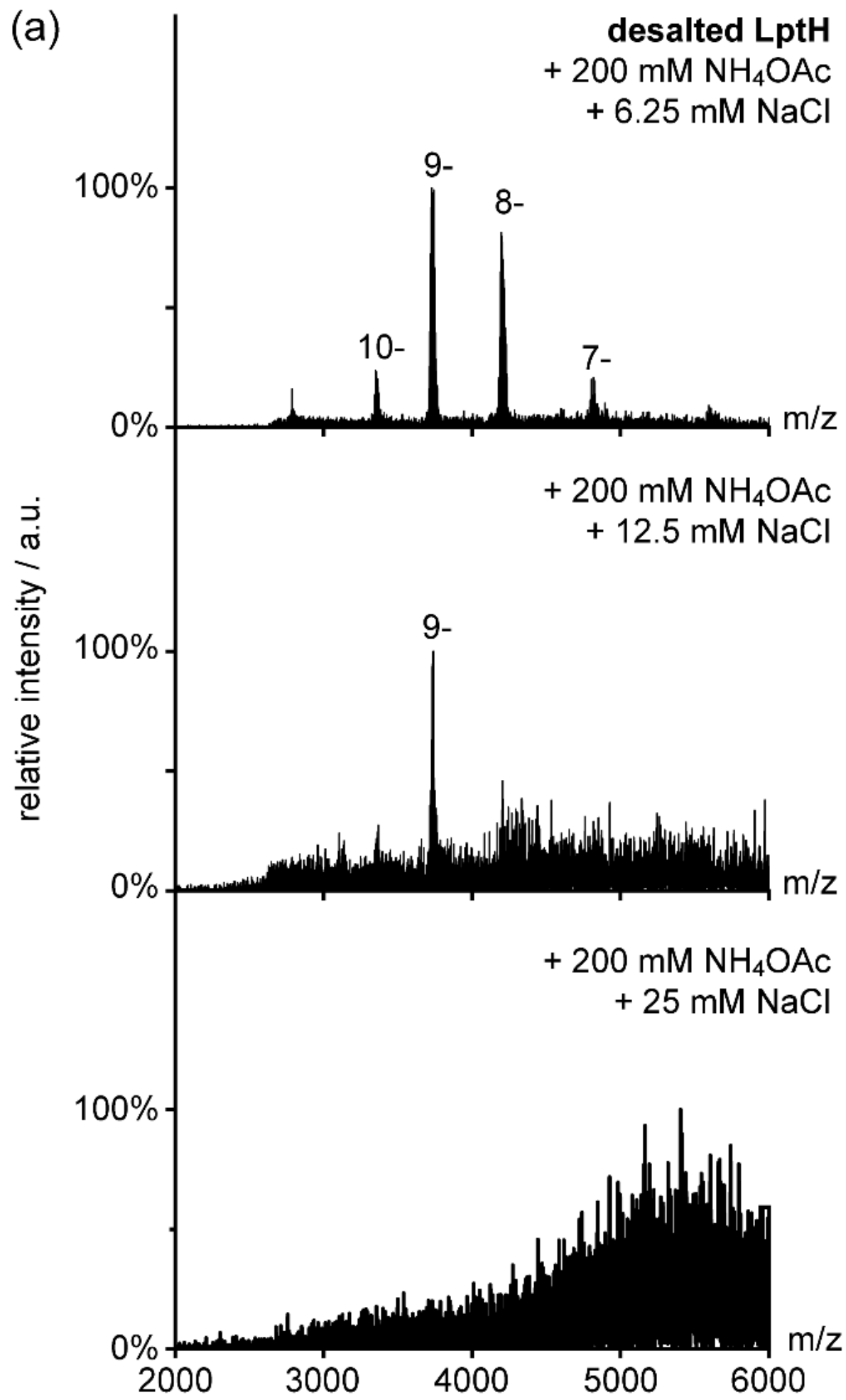

Figure S7. Sodium chloride tolerance in native MS of LptH with [G1]. (a) Mass spectra obtained from desalted LptH upon controlled addition of sodium chloride are compared with (b) mass spectra obtained upon addition of [G1].

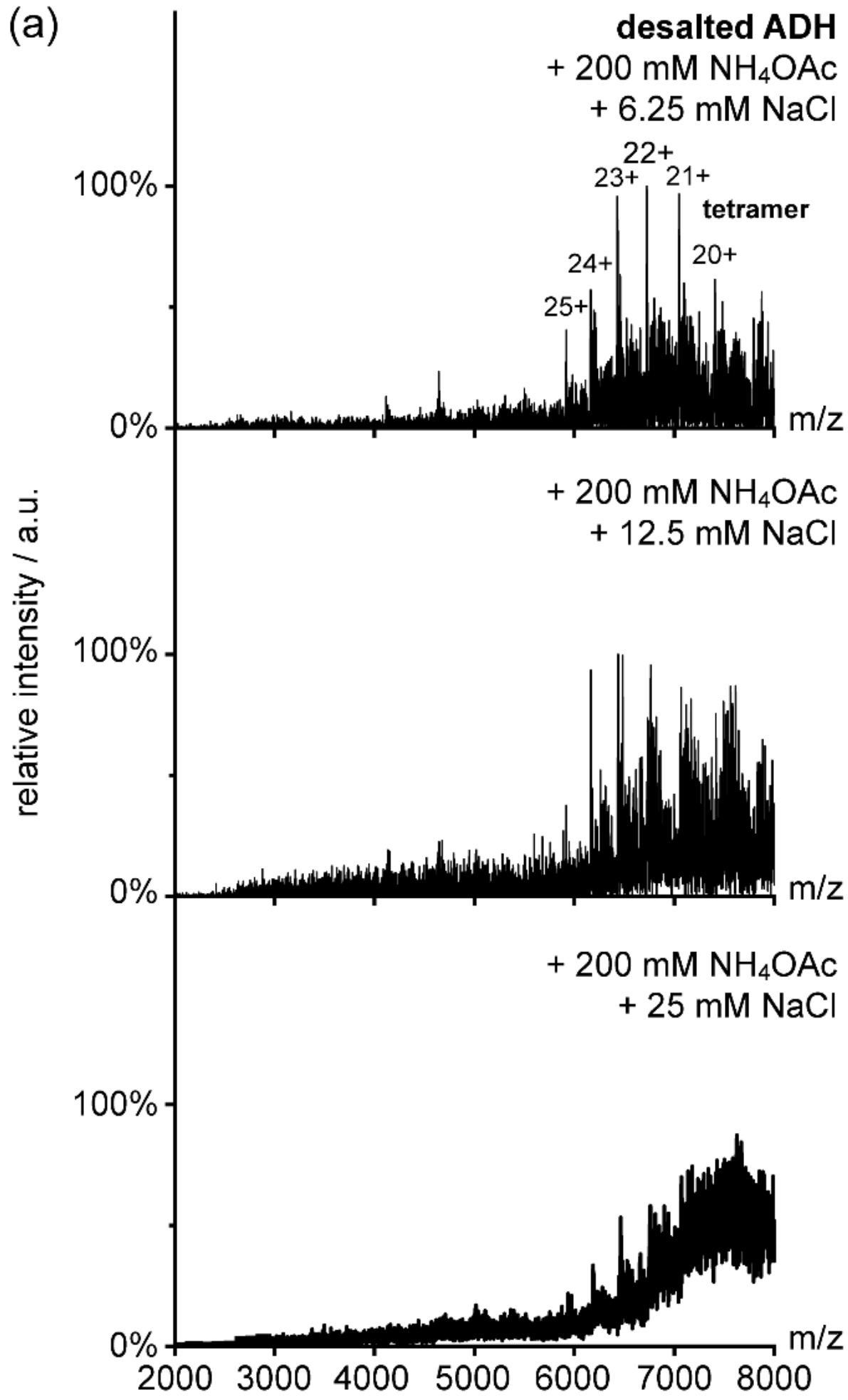

Figure S8. Sodium chloride tolerance in native MS of ADH with [G1]. (a) Mass spectra obtained from desalted ADH upon controlled addition of sodium chloride are compared with (b) mass spectra obtained upon addition of [G1].

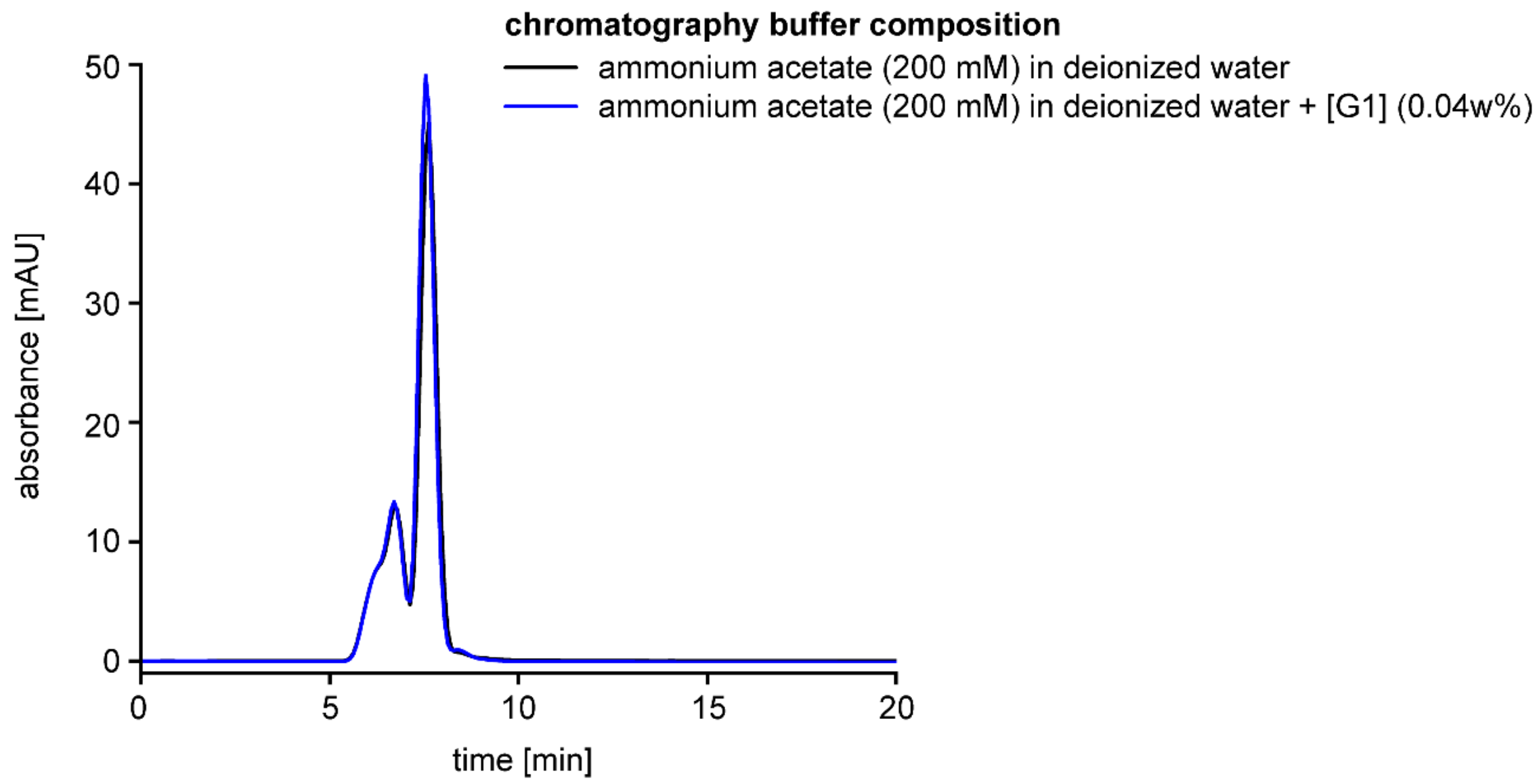

Figure S9. Size-exclusion chromatograms obtained from BSA in different chromatography buffer compositions. BSA was dissolved in the respective chromatography buffer systems prior to analysis.

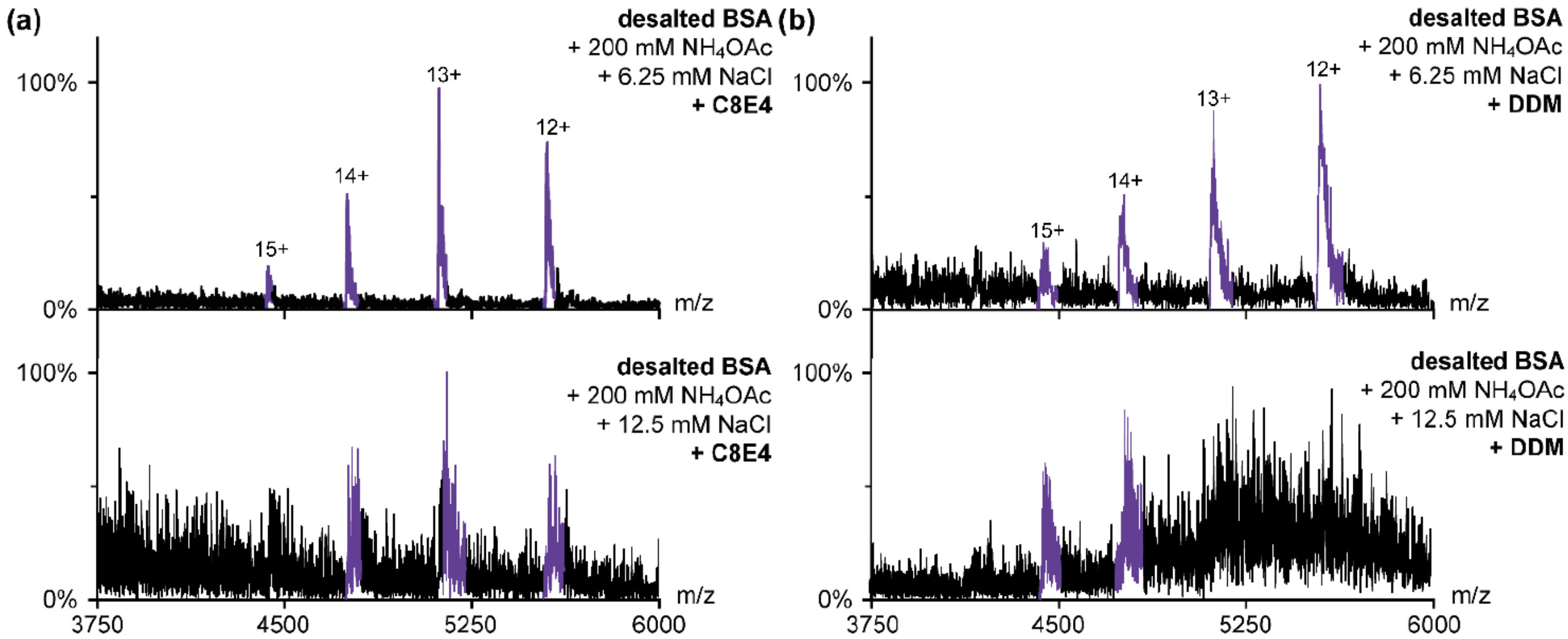

Figure S10. Sodium chloride tolerance in native MS of BSA with C_8E_4 and DDM. (a) Mass spectra obtained from desalted BSA upon controlled addition of sodium chloride and C_8E_4 are compared with (b) mass spectra obtained upon addition of DDM.

Supplementary Tables

Table S1. Diffusion coefficients of detergent (averaged from five signals) in dependence of the sodium chloride concentration (c_{NaCl}) and the hydrodynamic radii (R_h) calculated from Stokes-Einstein and the corrected hydrodynamic radius ($R_{h(\eta)}$) by the calculated dynamic viscosity of deuterated water (D_2O) with the salt concentration.

c_{NaCl} [mg·ml^{-1}]	diffusion coefficient [m^2·s^{-1}]	R_h [nm]	η_{D2O} [Pa·s]	$R_{h(\eta)}$ [nm]
2.5	6.882E-11	2.89	0.001098	2.89
5.0	6.782E-11	2.93	0.00110549	2.91
10	6.649E-11	2.99	0.00112077	2.93
20	6.231E-11	3.19	0.00115258	3.04
50	5.092E-11	3.91	0.00125887	3.41

Table S2. Protein-specific polarity, HCD collision energy and in-source trapping voltage used for the native MS analysis of soluble proteins in the presence of [G1] and NaCl. Other acquisition parameters common to all protein measurements are reported in the Methods section.

Protein	Instrument polarity	HCD collision energy [V]	In-source trapping [V]
Myoglobin	Positive	0	0
LptH	Negative	0	+100
BSA	Positive	70	0
ADH	Positive	0	-150
Pyruvate kinase	Positive	0	-150

Table S3. Summary of theoretical masses and observed masses determined by native MS under varying conditions.

Protein	[NaCl]	[G1]	Intact oligomer	Theoretical mass [Da]	Observed mass ± SD [Da]	Δmass [Da]
Myoglobin (holo)	unspecified (> 0 mM)	NO	monomer	17,568	17,565 ± 1	-3
Myoglobin (holo)	unspecified (> 0 mM)	YES	monomer	17,568	17,566 ± 1	-2
BSA	unspecified (> 0 mM)	NO	monomer	66,432	66,428 ± 1	-4
BSA	unspecified (> 0 mM)	YES	monomer	66,432	66,429 ± 1	-3
BSA	12.5 mM	NO	monomer	66,432	NS[a]	-
BSA	12.5 mM	YES	Monomer	66,432	66,493 ± 2	61
BSA	25 mM	NO	monomer	66,432	NS[a]	-
BSA	25 mM	YES	Monomer	66,432	66,508 ± 17	76
BSA	0 mM	YES	monomer	66,432	66,428 ± 2	-4
BSA + 1 x POPG	0 mM	YES	monomer	67,181	67,178 ± 1	-3
BSA	3.125 mM	YES	monomer	66,432	66,429 ± 1	-3
BSA+1 POPG	3.125 mM	YES	monomer	67,181	67,180 ± 3	-1
BSA	6.25 mM	YES	monomer	66,432	66,429 ± 1	-3
BSA + 1 x POPG	6.25 mM	YES	monomer	67,181	67,178 ± 1	-3
ADH	unknown (> 0 mM)	NO	tetramer	147,396	147,535 ± 17	139
ADH	unknown (> 0 mM)	YES	tetramer	147,396	147,511 ± 3	115
Pyruvate kinase	unknown (> 0 mM)	NO	tetramer	231,920	232,069 ± 22	149
Pyruvate kinase	unknown (> 0 mM)	YES	tetramer	231,920	231,998 ± 16	78
Myoglobin (holo)	6.25 mM	NO	monomer	17,568	17,735 ± 24	167
Myoglobin (holo)	12.5 mM	NO	monomer	17,568	NS	-
Myoglobin (holo)	25 mM	NO	monomer	17,568	NS	-
Myoglobin (holo)	6.25 mM	YES	monomer	17,568	17,587 ± 1	19
Myoglobin (holo)	12.5 mM	YES	monomer	17,568	17,608 ± 2	40
Myoglobin (holo)	25 mM	YES	monomer	17,568	17,588 ± 1	20
LptH	6.25 mM	NO	dimer	33,360	33,667 ± 43	307
LptH	12.5 mM	NO	dimer	33,360	33,614 ± 12	254
LptH	25 mM	NO	dimer	33,360	33,568 ± 15	202
LptH	6.25 mM	YES	dimer	33,360	33,506 ± 13	146
LptH	12.5 mM	YES	dimer	33,360	33,424 ± 1	64
LptH	25 mM	YES	dimer	33,360	33,424 ± 1	64

ADH	6.25 mM	NO	tetramer	147,396	147,936 ± 38	540
ADH	12.5 mM	NO	tetramer	147,396	NS	-
ADH	25 mM	NO	tetramer	147,396	NS	-
ADH	6.25 mM	YES	tetramer	147,396	147,657 ± 17	261
ADH	12.5 mM	YES	tetramer	147,396	147,788 ± 10	392
ADH	25 mM	YES	tetramer	147,396	147,819 ± 21	423

[a] NS: no resolved spectrum

Table S4. Signal-to-noise ratios (S/N) of charge-state peaks in raw native *m/z* spectra acquired in NaCl-containing solutions, comparing samples without detergent to those supplemented with dendritic detergent [G1]. S/N values were computed from the peak apex of the indicated charge state and a nearby peak-free noise window, and the fold-change reports the improvement upon [G1] addition.

Protein	[NaCl]	Selected charge state	S/N (no detergent)	S/N (G1)	Fold (G1/no detergent)
Myoglobin	unspecified (> 0 mM)	8+	348.0	4194.5	12.1
BSA	unspecified (> 0 mM)	15+	3452.7	17996.3	5.2
ADH	unspecified (> 0 mM)	24+	222.8	534.5	2.4
Pyruvate kinase	unspecified (> 0 mM)	32+	284.5	577.1	2.0
Myoglobin	6.25 mM	8+	32.1	555.9	17.3
Myoglobin	12.5 mM	8+	NS[a]	1788.5	-
Myoglobin	25 mM	8+	NS	238.2	-
LptH	6.25 mM	9-	250.6	700.2	2.8
LptH	12.5 mM	9-	24.5	81.6	3.3
LptH	25 mM	9-	NS	118.9	-
BSA	6.25 mM	15+	14.1	140.3	9.9
BSA	12.5 mM	15+	4.1	112.5	27.7
BSA	25 mM	15+	NS	31.6	-
ADH	6.25 mM	24+	16.1	313.3	19.5
ADH	12.5 mM	24+	10.5	37.8	3.6
ADH	25 mM	24+	NS	14.6	-

[a] NS: no resolved spectrum
